# Global slowness and increased intra-individual variability are key features of attentional deficits and cognitive fluctuations in post COVID-19 patients

**DOI:** 10.1038/s41598-022-17463-x

**Published:** 2022-07-30

**Authors:** Paola Ortelli, Francesco Benso, Davide Ferrazzoli, Ilaria Scarano, Leopold Saltuari, Luca Sebastianelli, Viviana Versace, Roberto Maestri

**Affiliations:** 1 Department of Neurorehabilitation, Hospital of Vipiteno (SABES-ASDAA) - Lehrkrankenhaus der Paracelsus Medizinischen Privatuniversität, Margarethenstr. 24, 39049 Vipiteno-Sterzing, BZ Italy; 2 Laboratory of Observational, Diagnosis and Education (ODFLab), Department of Psychology and Cognitive Science, University of Trento, 38068 Rovereto, Italy; 3 Department of Geriatrics, Memory Clinic, Hospital of Merano (SABES-ASDAA), BZ, Italy; 4 IRCCS Istituti Clinici Scientifici Maugeri, Montescano, Pavia Italy

**Keywords:** Psychology, Human behaviour

## Abstract

Fatigue, attentional deficits and cognitive fluctuations are the most characterizing symptoms of neurological involvement in Post COVID-19 syndrome (PCS). As the intraindividual variability (IIV) in cognitive performances has been recognized as a hallmark of brain-related disorders associated with cognitive deficits, it could be an interesting measure to elucidate the mechanisms subtending both the attentive impairment and the cognitive fluctuations in these patients. By referring to IIV analysis of Reaction Times (RTs), the present study aims to define the attentive impairment and its relation to fluctuations and fatigue, in patients suffering from Post COVID-19 neurological symptoms. 74 patients were enrolled. They underwent an extensive clinical and neuropsychological assessments, as well as computerized Sustained Attention and Stroop tasks. For studying IIV, RTs distributions of performances in computerized tasks were fitted with ex-Gaussian distribution, for obtaining the τ values. Finally, the Resting Motor Threshold (RMT) was also collected to estimate cortical excitability. 29 healthy volunteers served as controls. Patients showed poorer scores in Montreal Cognitive Assessment and higher RMT, in comparison with controls. In Sustained Attention Task, Mean, µ, σ and τ values were significantly higher in PCS patients (p value =  < 0.0001; 0.001; 0.018 and < 0.0001, respectively). Repeated measures ANOVA comparing the RTs mean in Stroop task within-subject and between-subjects revealed significant condition and group effect (p < 0.0001 both) and significant interaction (p = 0.005), indicating worst performances in patients. The mean of the derived interference value was significantly higher in PCS patients than in controls (p = 0.036). Patients suffering from PCS show deficits in attention, both in the sustained and executive components. Both high RTs means and high IIV subtend these deficits and could explain the often-complained cognitive fluctuations in this population.

## Introduction

About 30% of patients who recovered from the acute phase of SARS-CoV-2 infection (COVID-19) experience a disease state characterized by a plethora of long-term signs and symptoms that impact dramatically on daily living, working, and social life^1–5^. This lingering condition has been recognized as Post COVID-19 Syndrome (PCS)^1,6^. According to WHO, PCS occurs in individuals with a history of probable or confirmed SARS-CoV-2 infection, usually 3 months from the onset of COVID-19 with symptoms that last for at least 2 months and cannot be explained by an alternative diagnosis^7^. PCS symptoms may impressively fluctuate or relapse over time^4,7^. Fluctuations in the symptomatology are so frequent and strong, that many patients refer to the feeling to be “on a roller coaster”^4^. Millions of individuals suffer from PCS, regardless of their age or COVID-19 severity^2,8^.

Despite a tendency toward a progressive, slow remission and recovery^2,8,9^, the neurological symptoms of PCS last for a longer time, also in patients who suffered from asymptomatic infection or mild COVID-19^2,9^. Among the neurological manifestations, fatigue and cognitive difficulties affect more than 85% of PCS patients^9^. The mechanisms underlying these neurological manifestations are not completely understood. To deeply understand what subtend these symptoms and their characteristics, we studied patients reporting lingering fatigue and/or cognitive difficulties after severe and mild SARS-CoV-2 infection^10–12^. We found impairments in executive functions, specifically in the executive component of attention, thus confirming what has been previously found in PCS patients^2,9–13^. Executive attention is one of the main “top-down” functions regulated by the cognitive control network^14^. Attentional impairments may explain what happens in PCS patients, who often report feeling as fluctuating and cognitively blunted^3^. The neurophysiological evidence was suggestive of cortical hyperexcitability, as unveiled by increased resting motor threshold (RMT)^11,12^, a reliable estimation of cortical excitability^15–17^. These findings converge toward the hypothesis that impaired executive attention and central fatigue represent, at least in part, the minimum common denominator of the neurological manifestations in PCS. Nevertheless, little is known about the physiopathology of these conditions and how each other are connected.

Classical studies based on reaction times (RTs) tasks explored the link between “central” fatigue and attention^18,19^: patients with fatigue are slower and present increased Intraindividual variability (IIV) in RTs tasks exploring executive attention^20^. IIV reflects a transient within-person changes in cognitive and behavioral performances^21^ and their (in-)stability across time^22,23^. IIV is assumed to be related to different network states^20^ and has been recognized as a hallmark of many brain-related disorders associated with cognitive deficits^24^. Nowadays, in the neuroscience field, the study of IIV is worthy of interest, as it represents an attractive point of view for assessing the cognitive-behavioral impairment related to a given disease. Indeed, neuropsychological studies are in general primarily focused on differences among the means of performances obtained in the same experimental conditions by different groups. Instead, less interest has been given to the study of IIV and trial-by-trial fluctuations in the same clinical sample, both in terms of RTs and of day-by-day variations in cognitive performances. Nevertheless, this is a very interesting scientific and clinical aspects to address. In fact, the neglect of increased IIV when it represents a systematic feature in a given study population could represent an oversimplification of the adopted methodology, which may lead to misleading inferences^20^.

Given that, IIV measures may be useful for studying and understanding what lies behind the fluctuations’ phenomenon in PCS patients.

Different methods have been adopted to study IIV. For analyzing data collected with computerized RTs tasks, the ex-Gaussian analysis (based on the convolution of both Gaussian and an exponential distribution of the data) is considered the most reliable^25^. Specifically, the Gaussian component is thought to reflect sensory-motor and automatic processes, while the exponential component is thought to reflect central, attentive, and decision-related processes of executive attention^26^.

In this study, we aimed first at defining the attentive impairment in PCS patients and its relation to fluctuations. For this purpose, we collected RTs from two attentive computerized tasks and analyzed them using both Gaussian and ex-Gaussian approaches. Further, to interpret these data referring to the wider plethora of PCS symptoms, we also assessed collected subjective questionnaires to evaluate fatigue, mood, and sleep quality. Finally, RMTs were considered.

We hypothesize that the attention impairment relies on a global alteration of the executive attention system, which in turn is strictly related to fatigue and fluctuations in PCS patients.

## Results

Demographic data are reported in Table 1 and they show that the two groups are comparable in age, gender and education. Conversely, Table 2 depicts how, in comparison to healthy controls (HC), post COVID-19 Syndrome (PCS) patients obtained significantly lower Montreal Cognitive Assessment (MoCA) scores and showed higher RMTs.

**Table 1:** Comparison of demographic data between PCS-pt and HC.

	HC (29)	PCS-pt (74)	p HC-PCS-pt
Age	44.2 ± 14.5	48.4 ± 12.6	0.14
Gender	12.0 (41.4%)	21.0 (28.4%)	0.20
Education	14.8 ± 2.4	14.3 ± 2.7	0.48

Reported p values are from Mann–Whitney U-test for Age and Education and from the Chi square test for Gender.

*pt* patients, *HC* healthy controls, *PCS* post COVID-19 syndrome.

**Table 2:** Mann Whitney analysis for comparing MoCA-scores and RMT, between PCS-pt and HC.

	HC	PCS-pt	p HC-PCS-pt^a^
MoCA	27.6 ± 2.2	25.4 ± 2.9	**0.002**
RMTs (%)	44.7 ± 6.1	54 ± (16.5)	**0.015**

*MoCA* montreal cognitive assessment, *RMTs* resting motor thresholds, *pt* patients, *HC* healthy controls, *PCS* post COVID-19 syndrome.

Significant values are in [bold].

^a^Both comparisons remained significant after Benjamini–Hochberg adjustment.

Table 3 resumes the results of clinical assessments of 72 PCS patients (two patients refused to undergo the evaluation): they presented high levels of perceived fatigue, mild levels of depression, and poor quality of sleep.

**Table 3:** PCS patients’ clinical assessment: for each questionnaire, scale or index, in column the cut-off scores (which are indicated from the international literature) have been reported. The last column reports the percentage of patients having pathological scores.

	PCS-pt (72)	Cut-off	%
Time from PCS onset	126.4 ± (105.7)		
FSS	47.4 ± 12.2	Score ≤ 46	62.5
BDI II	16.5 ± 8.2	Score ≤ 12	72
PSQI	8.5 ± 4.0	Score ≤ 5	80.5

*PCS* post COVID-19 syndrome, *FSS* fatigue severity Scale, *BDI II* beck depression inventory II, *PSQI* Pittsburgh sleep quality index.

The mean value and the value of the three parameters µ, σ and derived from ex-Gaussian fitting for Sustained Attention Task (SAT) in PCS patients and HC are reported in Table 4. All considered parameters were significantly higher in PCS patients than in HC.

**Table 4:** Mann Whitney analysis for comparing parameters of Sustained Attention Task (SAT) RTs.

	HC (29)	PCS-pt (74)	p HC-PCS-pt^a^
SAT mean	323.5 ± 37.2	428.6 ± 168.9	**< 0.0001**
SAT µ	262.7 ± 26.3	296.6 ± 64.2	**0.001**
SAT σ	21.1 ± 9.5	32.4 ± 38.0	**0.018**
SAT τ	62.3 ± 18.1	132.0 ± 117.4	**< 0.0001**

Significant values are in [bold].

^a^All comparisons remained significant after the Benjamini–Hochberg adjustment.

The Stroop task was correctly completed by 69 patients. The Stroop assessment in the remaining 5 patients was interrupted because of difficulties in understanding the task rules. The mean value of Stroop Task in Word Color Naming (WCN) and Color Naming (CN) conditions and Interference (I-ST) are reported in Table 5. Again, the values were significantly higher in PCS patients than in HC.

**Table 5:** Results by Mann Whitney analysis for comparing parameters of Stroop Task (ST) RTs, for conditions (WCN–CN) and derived RTs (I).

	HC (29)	PCS-pt (69)	p HC-PCS-pt^a^
WCN-ST mean	858.5 ± 150.4	1156.5 ± 391.2	**< 0.0001**
CN-ST mean	726.9 ± 110.8	879.3 ± 183.4	**< 0.0001**
I-ST mean	131.6 ± 104.1	277.2 ± 260.7	**0.036**

*WCN* word color naming, *CN* color naming, *I* interference, *pt* patients.

Significant values are in [bold].

^a^All comparisons remained significant after the Benjamini–Hochberg adjustment.

**Figure 1:**
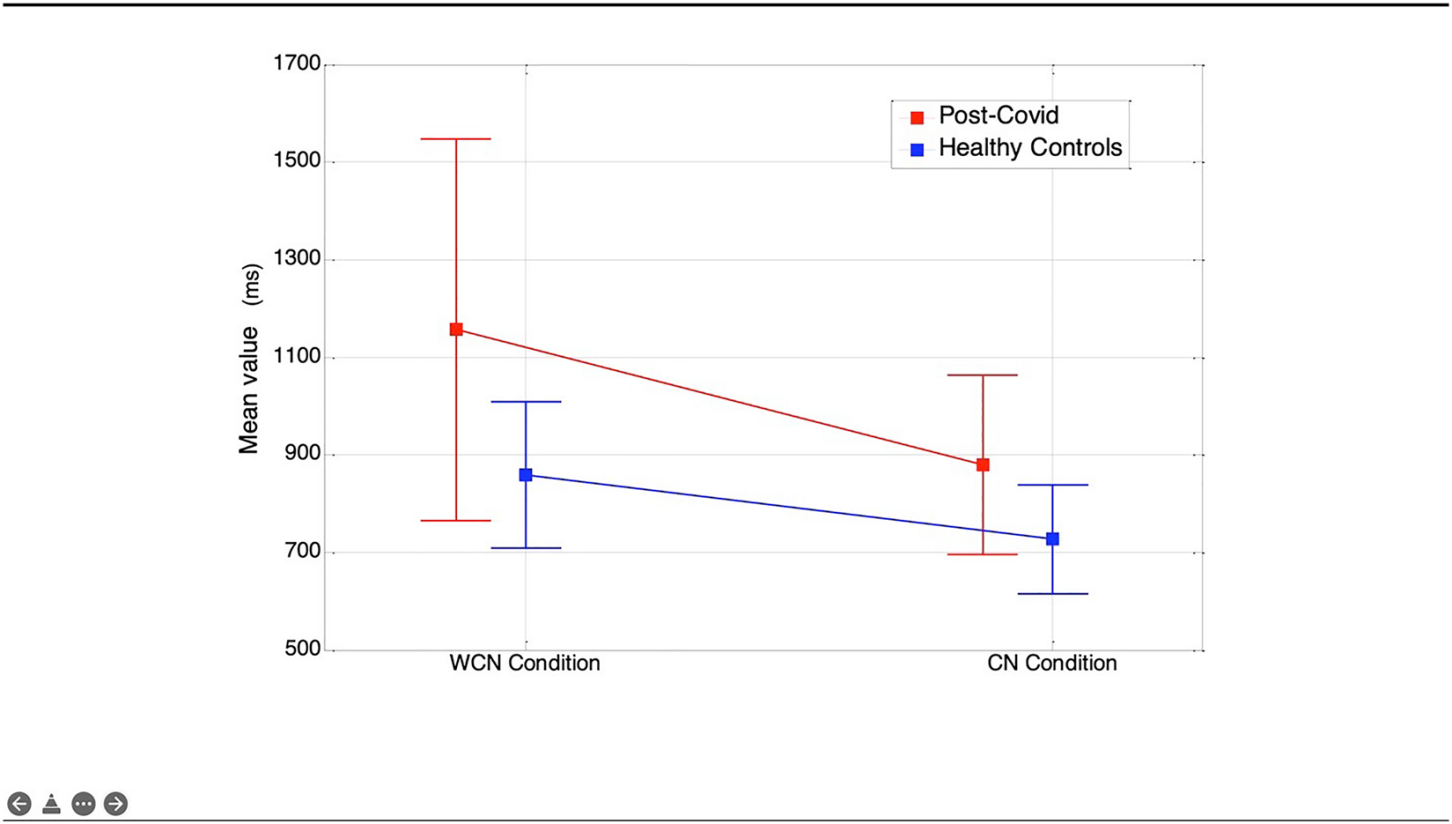
Mean value of RTs during stroop task in WCN and CN conditions, in PCS-pt and HC.

Repeated measures ANOVA comparing the mean in the two conditions Stroop Task WCN and CN (within-subject) in the two groups (PCS patients and HC) revealed significant condition and group effect (F(1, 96) = 66.5 and F(1, 96) = 17.7, respectively; p < 0.0001 both) and significant interaction (F(1, 96) = 8.4; p = 0.005). A graphical representation of this relationship is given in Fig. 1. Finally, correlation coefficients (Spearman’s r) for PCS patients only between sustained attention tasks (SAT mean and SAT τ) and MoCA, Fatigue Severity Scale (FSS), Beck Depression Inventory (BDI-II), Pittsburgh Sleep Quality Index (PSQI), and time from the onset are reported in Table 6 (first two columns). In addition, correlation coefficients (Spearman’s r) between I-ST mean and MoCA, FSS, BDI-II, PSQI, and time from the onset are reported in the last column of Table 6.

**Table 6:** Results by Spearman r analysis (r and p-value), for studying the relation of SAT RTs and derived I-ST with clinical and cognitive global scores.

	SAT mean	SAT τ	I-ST mean
MoCA	− 0.08 (p = 0.52)	− 0.05 (p = 0.66)	**− 0.28 (p = 0.021)**
FSS	**0.32 (p = 0.006)**	**0.28 (p = 0.018)**	− 0.01 (p = 0.95)
BDI II	**0.33 (p = 0.005)**	**0.31 (p = 0.007)**	− 0.08 (p = 0.54)
PSQI	0.16 (p = 0.17)	0.20 (p = 0.09)	− 0.12 (p = 0.35)
Time from PCS onset	− 0.06 (p = 0.61)	− 0.05 (p = 0.67)	− 0.01 (p = 0.93)

*MoCA* montreal cognitive assessment, *FSS* fatigue severity Scale, *BDI II* beck depression inventory-II, *PSQI* Pittsburgh Sleep Quality Index, *PCS* post COVID-19 syndrome.

Significant values are in [bold].

## Discussion

**Figure 2:**
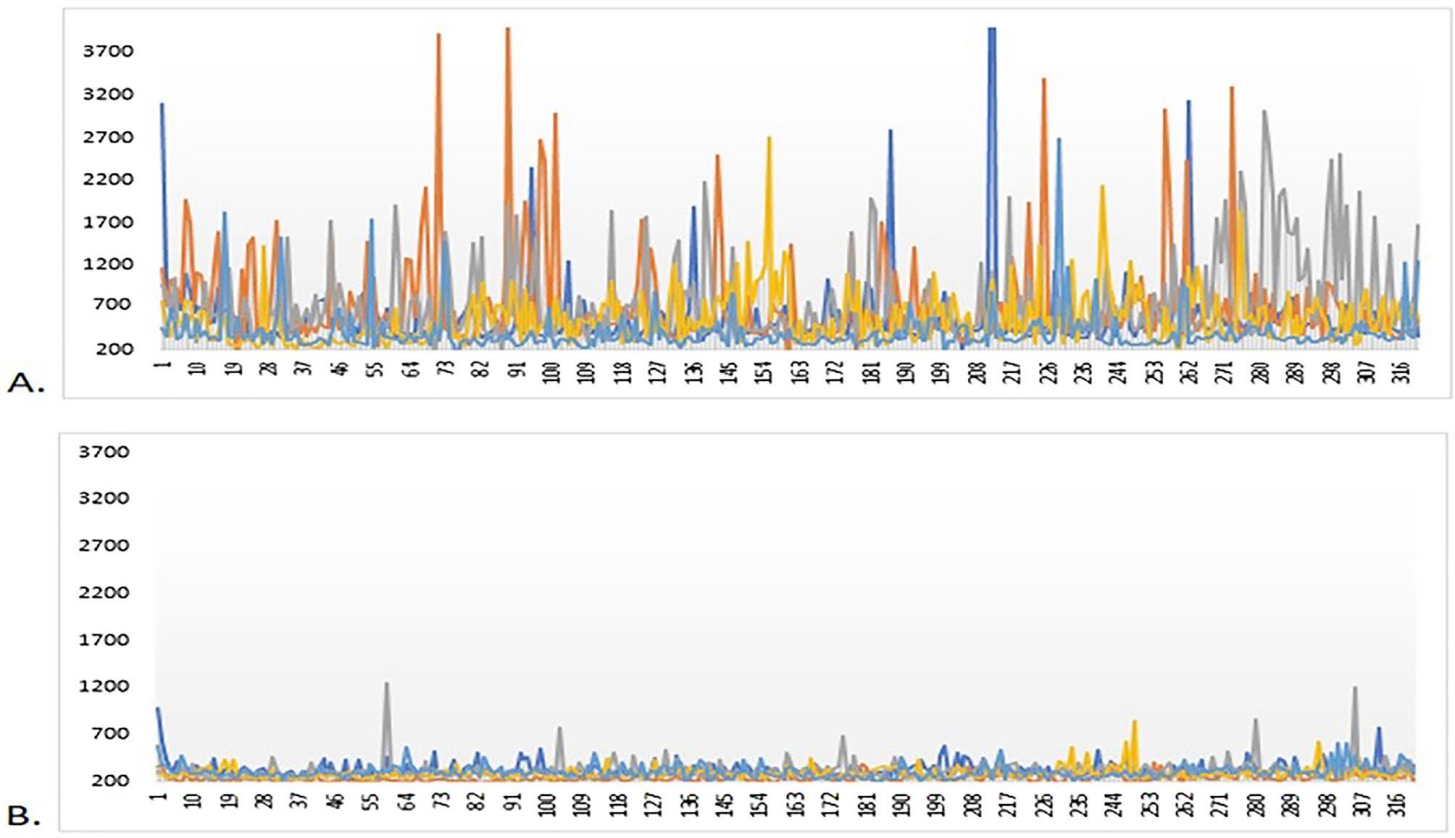
Comparison between variability of RTs in 5 PCS patients (**A**) and 5 HC (**B**).

To the best of our knowledge, this study represents a starting point to better understanding the attentional alterations described in PCS patients with fatigue and symptomatic fluctuations. Studying sustained attention and interference inhibition, we observed a high slowness in RTs. In accordance with previous data^13^, these results indicate dysfunctions in the executive attention system network. Both, global slowdown and dysexecutive control contribute to the disruption of the executive attention system. The global slowdown is evident from the mean of RTs, which was higher compared to that of HC, in all task conditions. Dysexecutive control is highlighted by the IIV analysis of RTs in Sustained Attention Task (SAT) and by the mean analysis of derived I-ST RTs in ST. The strong IIV in SAT could be considered the first objective evidence of fluctuations, often reported by PCS patients (see Fig. 2). PCS patients presented higher RMTs, thus indicating cortical hypoexcitability^27^. Contextually, they subjectively complained of high levels of fatigue, poor quality of sleep, and mild depressive symptoms.

PCS is a poorly-understood disorder characterized by multisystem symptoms^28^. Among them, fatigue and cognitive difficulties are the most frequently complained, other than the most persistent^2,9,29^. It is known from the clinical practice that these symptoms may fluctuate. Nonetheless, the nature of these fluctuations is largely unclear^4^. The main PCS cluster of symptoms, represented by fatigue, cognitive dysfunctions, and fluctuations, impacts seriously on quality of life^2^.

Similar symptoms, often in association with sleep disturbances and mood alterations, have been previously described in numerous neurological or psychiatric diseases, such as Parkinson´s disease, chronic fatigue syndrome (CFS), multiple sclerosis (MS), and as stroke complications^30,31^. In PCS, cognitive dysfunctions and fluctuations seem to be properly linked to pathological fatigue^31,32^.

No association emerged between SAT RTs and PSQI, while we observed a strong association among SAT RTs mean (evaluating the sustained attention) with FSS (indicating high levels of fatigue) and BDI-II (showing the presence of a mild depressive syndrome). These results allow arguing that global slowness, arising from higher RTs and higher IIV, expresses alterations of the attention system^33^.

PCS patients showed higher derived I-ST RTs mean in comparison with HC. This task is a computerized tool based on classical Stroop Effect, in which the time spent elaborating a specific feature of a given stimulus increases when automatic and interfering elaborations of other features of the same stimulus must be suppressed. The ability to inhibit interference is a key component of executive attention. We also observed an association between derived I-ST RTs mean and MoCA scores. The latter is an interesting result, as it provides a possible explanation for the poor performances the PCS patients presented in this global cognition test, which requires spared executive functions for achieving good performances.

Finally, the analysis of both the “derived” I-ST RTs mean and the SAT RTs IIV suggests that the executive control system is likewise impaired. The direct implications can be easily understood, as the executive control system is aimed at maintaining the goal during cognitive tasks across time, and at controlling competing pathways during data elaboration^14^.

The presumed impairment of the executive attention system in PCS is also strengthened by the higher SAT RTs IIV. Indeed, the increase in RTs IIV is defined to be consistent with a breakdown in the attentional executive system^26^. More specifically, increased IIV has been linked to frontal-cortex-mediated processes, such as attentional lapses and fluctuations in executive control^20^. Previous studies^34,35^ indicate that high IIV is related to white matter hyperintensity in the frontal lobe. Consistently, other authors^36^ found that patients with frontal lobe lesions showed increased inconsistency in task performances.

Increased IIV is strictly associated with the fatigue reported by our population: this relation is supported by previous studies highlighting greater IIV both in CFS and MS patients complaining of pathological fatigue and depressive symptoms^37–40^. Coherently, RMTs in PCS patients were higher in comparison with HC, as previously described in fatigued patients presenting cortical hypoexcitability^15–17,41^.

High RMTs are reported in other neurological conditions causing fatigue^15,42^.

This is the reason why the detection of parameters indicative of altered excitability is considered an early neurophysiological marker of pathological cortical conditions^43^.

In conclusion, we observed a very high IIV (Fig. 2). IIV reflects both trial-by-trial fluctuations and day-to-day variations in cognitive performances^20^. IIV in response time provides information about attention abilities beyond accuracy and mean RTs^44^. It is considered an endophenotype for a wide range of clinical disorders and a general marker of neurological health or maladaptation^45^. As a matter of fact, response stability in adults is supported by the central executive and the salience networks^46,47^. Therefore, changes in IIV could be considered as the expression of changes in different brain network states.

This study presents such limitations that need to be acknowledged. First of all, it is difficult to address whether PCS patients manifest slower RTs due sole complications of COVID-19 instead of other confounding factors. Further studies will need to better understand this aspect.

To conclude, the present study unveils an impairment of executive attention system in patients who recovered from SARS-CoV-2 infection and complaining of PCS with lingering cognitive difficulties, fatigue and fluctuations. These deficits involve both sustained attention and interference. In light of previous evidence, this study supports the hypothesis that increased IIV in computerized tasks and greater sensibility to interference reflects the neural origin of this complex and lingering condition.

## Materials and methods

Between March and July 2021, we enrolled at the Post COVID-19 outpatient clinic of the Department of Neurorehabilitation (Hospital of Vipiteno, Vipiteno-Sterzing, Italy) 74 PCS patients (mean age 48.4 years; mean education level 14.8 years). The time elapsed between the disease onset and the study participation was calculated.

Inclusion criteria were: (a) the previous diagnosis of SARS-CoV-2 infection confirmed by PCR testing of a nasopharyngeal swab; (b) consequent infection resolution defined by two consecutively negative PCR tests separated by ≥ 1 day; (c) mild form of COVID-19 (symptoms may include fever, cough, sore throat, malaise, myalgias, gastrointestinal symptoms, anorexia, nausea, and diarrhoea, anosmia and ageusia); (d) complaining of cognitive difficulties and/or sense of fatigue, persisting after COVID-19 resolution.

Exclusion criteria: (a) prior or concurrent diagnosis of other neurologic, psychiatric, endocrine, metabolic, or cardiopulmonary conditions; (b) clinical and/or radiological evidence of COVID-19 related-pneumonia during the active phase of the disease; (c) anemia; (d) pharmacological treatment with corticosteroids, antihistaminic, antihypertensive, diuretic, antidepressant, anxiolytic or hypnotic drugs at the time of the study.

29 sex and age-matched healthy controls (HC) without any evidence of SARS-CoV-2 infection (mean age 48.4 years; mean education level 14.3 years) underwent the same assessment.

The study was approved by the local Ethics Committee (“Comitato Etico del Comprensorio Sanitario di Bolzano”) (65-2020) and was in accordance with the code of Ethics of the World Medical Association (Declaration of Helsinki, 1967). All participants signed an informed written consent form for the use of their clinical data for scientific purposes.

### Clinical assessment

Demographic data, medical history, and previous PCR tests were collected. All patients and HC underwent an extensive neuropsychological evaluation.

#### Fatigue assessment

Perceived fatigue was assessed in both PCS patients and HC through the Fatigue Severity Scale (FSS)^48^, referring to the last week previous to the evaluation. FSS consists of 9 items exploring the interference of fatigue with certain activities of daily living and rates its severity according to a 7-point self-report scale (1 = “strongly disagree”; 7 = “strongly agree”)^49^.

#### Mood and quality of sleep assessment

The Beck Depression Inventory-II (BDI-II) is a 21-question multiple-choice self-report inventory for measuring the severity of depression^50^. Each answer is scored on a scale value of 0 to 3. Higher total scores indicate more severe depressive symptoms.

The Pittsburgh Sleep Quality Index (PSQI) is a self-report questionnaire that assesses the quality of sleep over a 1-month interval^51^. The measure consists of 19 individual items, creating 7 components that produce one global score.

#### Assessment of global cognition

Global cognition was evaluated through the Montreal Cognitive Assessment (MoCA)^52^.

### Neurophysiological assessment

The patients enrolled in this study were part of a larger cohort of subjects who underwent different types of neurophysiological investigations. Some of these investigations included the use of transcranial magnetic stimulation (TMS) and presented as a common denominator the RMT calculation, which is considered the stimulus intensity that causes a “minimum motor response” in a resting muscle during single TMS pulses applied over the “motor hotspot”^53^.

During the TMS experiments, the patients were sitting comfortably in an armchair with their eyes open. TMS was delivered over the dominant primary motor hand area through a tangentially oriented 7 cm figure-of-eight coil connected via a Bistim module with two Magstim 200 stimulators (Magstim Company, Whitland, Dyfed, UK) and placed over the optimal site for eliciting motor evoked potentials (MEPs) in the contralateral first dorsal interosseous (FDI) muscle. The coil position was continuously monitored during the entire experiment.

RMT was defined as the lowest TMS intensity (expressed in percentage of the maximum stimulator output) that evoked MEPs of at least 50 µV peak-to-peak amplitude in five of ten successive trials^54^.

### Computerized assessment of attentional system

All participants were tested in a laboratory setting, with constant artificial light, and without auditory interference. Reaction times (RT) in tasks evaluating sustained and executive attention were assessed with randomized computer-controlled RT paradigms (implemented with SuperLab 5^®^)^55^. Patients underwent two computerized attentive tasks. Participants were asked to sit in front of a 16-in. color monitor (display resolution 3072 × 1920 pixels, 226 dpi) at a comfortable distance. For each task, participants first read the instructions and performed two training sessions: the first one to acquire confidence with the keyboard and the buttons associated with a specific response; the second session needed to make subjects confident with the experiment and to avoid any bias related to learning effects.

Participants were instructed to place their preferred hand on the computer keyboard. They had to fix the screen and press the respective response key at the appearance of a target stimulus. Between stimuli, participants had to keep their “response hand” on the keyboard. Each experimental task consisted of different blocks of trials: Participants were allowed to take a rest between two consecutive blocks.

- The Sustained Attention Task (SAT) evaluates the speed with which subjects respond to a specific environmental stimulus. The experimental task consisted of 320 trials divided in two blocks. Participants had to press a response button as quickly as possible after the appearance of a target that disappeared after striking the response key. Targets consisted of a black circle that appeared always in the center of a white screen at randomized intervals (Interval Inter Stimulus—ISI—1000–1500–2000–2500 ms).
- The Stroop Task (ST) assesses the participant’s ability to inhibit cognitive interference, which occurs when automated processing of a stimulus feature affects the simultaneous processing of another attribute of the same stimulus.^56^ The task consisted of six blocks, for a total of 216 trials, divided into two conditions (Word Color Naming—WCN—and Color Naming—CN—see above). Participants had to press corresponding keys related to differently colored circles (CN) as fast as possible. In WCN (“interference condition”) names of colors were printed in inconsistent colors, and subjects had to press a key corresponding to the color of the ink instead of the word’s meaning. Therefore, participants had to perform a less automated task (naming ink color) while inhibiting the interference arising from a more automated task (reading the word). In each condition, targets appeared in the center of the screen and disappeared after striking a key. Finally, the difference between WCN and CN is considered an expression of the Interference component^57^.

RTs shorter than 100 ms were deemed outliers, excluded from analysis and their number was noted.

For each task, the mean value was computed and RTs distributions were fit with ex-Gaussian distribution using maximum likelihood estimation ad a bounded Simplex algorithm^58^. From the resulting ex‐Gaussian function three parameters, μ, σ, and τ were obtained: the first two parameters (μ and σ) correspond to the mean and standard deviation of the estimated Gaussian component (sensory-motor and automatic processes), the third parameter (τ) is the mean of the estimated exponential component (central, attentive and decision-related processes of executive attention).

### Statistical analysis

The central tendency and dispersion of continuous variables were reported as mean ± SD. Descriptive statistics for categorical variables were reported as N (percent frequency). Between-group comparisons were carried out by non-parametric Mann–Whitney U-test and by the Chi-square test for continuous and categorical variables respectively. Repeated measures analysis of variance (rm-ANOVA) was used to compare results for the Stroop Task in the two conditions (WCN Stroop Task and CN Stroop Task) in the two groups (PCS patients and HC), with repeated measures in the condition factor. The association between couples of variables was assessed by the Spearman rs correlation coefficient.

All tests were two-tailed. A p-value < 0.05 was considered statistically significant.

When appropriate, the false discovery rate (FDR) was controlled at 5% using the Benjamini–Hochberg method. All analyses were carried out using the SAS/STAT statistical package, release 9.4 (SAS Institute Inc., Cary, NC, USA).

## Data Availability

The data that support the findings of this study are available on request from the corresponding author. The data are not publicly available due to privacy or ethical restrictions.

## Code availability

All source codes and models are available for consultation by the authors’ permission.
